# Ureteral stent complications – experience on 50,000 procedures

**DOI:** 10.25122/jml-2021-0352

**Published:** 2021

**Authors:** Petrișor Geavlete, Dragos Georgescu, Razvan Mulțescu, Florin Stanescu, Cosmin Cozma, Bogdan Geavlete

**Affiliations:** 1. Department of Urology, Sfantul Ioan Emergency Clinical Hospital, Bucharest, Romania; 2. Department of Urology, Carol Davila University of Medicine and Pharmacy, Bucharest, Romania

**Keywords:** double J stent, complications, urinary tract infection, stent encrustation, stent migration

## Abstract

Double J stent is an essential tool in urology, being a basic part of many urological procedures. However, some issues related to their use still occur. Our study aimed to evaluate an important number of procedures, the complications of ureteral stents, and their prevention and treatment retrospectively. We evaluate 50,000 procedures performed between 1996 and 2021 on 36,688 patients. According to the stenting duration, the cases were divided into short-term (less than 6 weeks – 34,213 procedures), respectively long-term stenting (more than 6 weeks – 15,757 procedures). The indications of stenting for both groups were noted. The total number of complications was 41,369. We encountered 153 cases (0.3%) of JJ stent malposition, of which 3 cases were into the retroperitoneum, one case with parenchymal perforation and hematoma. Considering the double J migrations, we found proximal migration in 427 cases (0.9%) and distal double J migrations in 352 (0.7%) cases. The obstruction of the ureteral stent, causing inefficient drainage, was encountered in 925 cases, while irritative bladder symptoms occurred in 16,326 cases (32.7%). Hematuria was observed in 5,213 cases, in 7 cases blood transfusion being necessary. Urinary tract infection was diagnosed in 7,436 cases (14.8%). Stent encrustation and calcification occurred in 832 cases, while stent fragmentation was noted in 52 cases. Double J stent complications should be promptly evaluated and treated. Encrustation and stone formation in forgotten stents often lead to serious complications and should be managed with stent removal and combined endourological techniques.

## Introduction

First reported by Zimskind *et al.* in 1967, double J (DJ) ureteral stent is an important therapeutic tool in endourology. Gibbons described the auto-retention ureteral stent in 1976, and, afterward, Finney introduced the term “double J stent” in 1978. The double J stents are an important part of many urological procedures, such as endoscopic or open surgery for retroperitoneal tumors of fibrosis, ureteral strictures, or treatment for reno-ureteral stones [1]. In addition, the stents can also be placed after iatrogenic injuries of the ureter to protect and reveal the ureter in complex abdominal surgery [2].

However, despite the progress in materials and design, some problems regarding their use still develop. Since the stent is a foreign body, it may cause patient discomfort, bacterial colonization, hematuria, irritative voiding symptoms, and deposition of urine constituents. These issues profoundly affect the therapeutic outcome and patient’s quality of life. Our objective was to review the indications for using ureteral stents, identifying their major connecting symptoms, complications, and prevention.

## Material and Methods

In the present study, we evaluated 50,000 procedures performed between 1996 and 2021 on 36,688 patients retrospectively. Among these, 44,523 of the procedures were unilateral, and 5,477 were bilateral. Forty-three percent of the patients were men, and 57% were female.

According to the stenting duration, the cases were divided into short-term, respectively long-term stenting. We considered short-term stenting the indwelling catheters placed for up to 6 weeks and long-term stenting for double J for more than 6 weeks. According to these criteria, 34,213 procedures were performed for short-term drainage in 32,456 patients. The indications for short-term stenting are depicted in Table 1.

**Table 1.** Indications for short-term stenting.

Indications	Procedures
**Post-operative**	31,226
**Pre-ureteroscopy**	629
**Pregnant women**	456
**Prevention of ureteral trauma**	42
**Obstructive pyelonephritis**	1,749
**Ureteral lesions**	74
**Others**	37

The procedures for postoperative temporary drainage are represented by stenting post-ureteroscopy for lithiasis, conservative treatment for upper urinary tract carcinomas, caliceal diverticula, endopyelotomy, or following open surgery, including pyeloplasty or ureteral segmental resection. In 42 cases, the double J stent was placed prior to gynecological or abdominal surgery to avoid ureteral trauma. The long-term stenting is represented by 15,757 procedures performed on 4,232 patients. Double J placement was performed for extrinsic ureteral stenosis, whether it was by benign compression (retroperitoneal fibrosis, fibrosis post-irradiation) or malignant (obstruction by extrinsic tumoral compression or invasion), or in patients with lithiasis or intrinsic ureteral stenosis with contraindications for stone removal or definitive treatment of the diseases (Table 2).

**Table 2.** Indications for long-term stenting.

**Indications**	**Procedures**
**Benign extrinsic fibrosis**	4,924
**Malignant extrinsic fibrosis**	9,852
**Ureteral stenosis**	315
**Lithiasis**	306
**Other**	360

## Results

Ureteral stent complications were represented by stent malposition, migration, irritative bladder symptoms, lumbar pain, hematuria and urinary tract infections, encrustation, and stent fragmentation. We encountered 41,369 complications. According to the Clavien-Dindo scale, 19,502 (47.1%) were grade I, 18,558 grade II (44.8%), 3,266 (7.9%) grade IIIA, 35 grade IIIB, 5 grade IV, and 3 grade V.

We found 153 cases (0.3%) of double J stent malposition, of which 3 cases were into the retroperitoneum, one case with parenchymal perforation and hematoma, and 149 cases placed with the proximal end into the ureter. All cases were classified IIIA according to the Clavien-Dindo scale, endoscopic replacement being performed, 7 of them requiring ureteroscopic approach and other two percutaneous nephrostomies. In what concerns the double J migrations, proximal migration occurred in 427 cases (0.9%) of the cases, 241 cases (0.7%) among the short-term stenting, and 186 (1.2%) among long-term stentings. Distal double J migrations were encountered in 352 (0.7%) cases, 145 in short-term and 207 in long-term stenting. The obstruction of the ureteral stent, causing inefficient drainage, was encountered in 925 cases (352 in patients with short-term stenting and 573 in patients with long-term stenting). Irritative bladder symptoms occurred in 16,326 cases (32.7%). In 2,354 cases, the symptoms were managed with specific medication (grade II – Clavien-Dindo), and in 37 cases, stent removal was necessary. Lumbar pain was noted in 9653 cases (19.3%), most of them due to vesicoureteral reflux. Stent removal was imposed in 42 de cases, while temporary bladder catheterization was performed in 6521 cases. All other cases were managed conservatively. Hematuria occurred in 5,213 cases, 7 cases requiring blood transfusion. From these cases, 4,887 occurred in patients with short-term stenting, the majority post-procedural. In these cases, hematuria could be secondary to the ureteroscopy. Urinary tract infection was diagnosed in 7436 cases (14.8%). Stent encrustation and calcification occurred in 832 cases, most in patients with long-term stenting (810 cases). In Table 3 we described all complications of the double-J ureteral stents. According to the site of encrustation/calcification, the cases were classified in:

• Bladder loop encrustation/calcification – 432 cases;
• Proximal loop calcification – 26 cases;
• Bladder and renal loop calcifications – 235 cases;
• Ureteral calcification – 2 cases;
• Bladder loop and ureteral calcifications – 25 cases;
• Entire stent encrustation/calcification – 112 cases.

**Table 3.** Double-J ureteral stent complications.

**Complication**	**Total**	**Short-term stenting**	**Long-term stenting**
**Stent malpositioning**	153	N/A	N/A
**Proximal stent migration**	427	241	186
**Distal stent migration**	352	145	207
**Stent obstruction **	925	352	573
**Irritative bladder symptoms**	16,326	N/A	N/A
**Lumbar pain**	9,653	7,852	1,801
**Hematuria**	5,213	4,887	326
**Urinary tract infection**	7, 436	3,256	4,180
**Encrustation and calcification**	832	12	810
**Stent fragmentation**	52	0	52

The treatment alternatives were represented by:

• Simple double J stent removal – 241 cases;
• Lithotripsy of the bladder loop calcifications – 324 cases;
• Lithotripsy of the bladder loop calcifications + PNL – 129 cases;
• Lithotripsy of the bladder loop calcification + retrograde ureteroscopy – 115 cases;
• PCNL – 11 cases;
• Retrograde ureteroscopy – 12 cases.

## Discussion

Double J ureteral stents represent a valuable tool in the urological practice. Depending on the indication, the stent could be placed temporarily or permanently. However, in the case of long-term stenting, regular replacements are necessary to prevent complications associated with prolonged indwelling times. Therefore, stents are usually replaced after 3 to 6 months [3]. Urologists increasingly use indwelling ureteral stents for urine diversion, ureteral blockage alleviation, and postoperative drainage, which has increased difficulties associated with their usage [4]. There are no established best practices for dealing with these issues. Due to the lack of a perfect stent, we are faced with issues such as stent migration, blockage, encrustation, fragmentation, and stone formation [4]. Different types of ureteral stents and biomaterials have been developed in recent years to reduce these negative effects. The ideal stent should be simple to insert, relieve obstruction, allow enough urine flow, resist incrustation and infections, be chemically stable, and not cause side effects. As a result, it must have high tensile strength, a low friction coefficient, and be self-holding [4]. Early complications, such as dysuria, stomach pain, and hematuria, can occur less than four weeks after the insertion, while late complications, such as migration, blockage, calcification, and urinary tract infection (UTI), can develop more than four weeks after the insertion [5].

### Malpositioning

Double J malposition represents an important intraoperative complication and is responsible for further incidents. In our study, the most common was the placement of the proximal end into the ureter, but we also experienced 3 cases with placement into retroperitoneum, one case with parenchymal perforation and hematoma. Renal parenchymal perforation is rare but life-threatening [6]. A malpositioning complication like intravascular migration of double J stent, ureteroarterial fistula, hemoperitoneum, or knotted stent has been reported as very rare [7].

### Migration 

Migration of double J stents is a known complication, and it can occur proximally into the pyelocaliceal system or ureter and distally towards the bladder. In our study, proximal double J migration was rarely encountered (0.9% of all cases), similar to literature data, occurring in 0.6% to 3.5% of all cases [8].

The incidence of stent migration reported by other authors was 5.8% [9]. According to the literature, increased stone diameter and severity of hydronephrosis may assist migration by reducing stent attachment [10]. Other studies determined that the lack of size of the double J stent affects stent migration, the bladder curl being inadequate to maintain the position of the stent. Therefore, the distance between the ureteropelvic and ureterovesical junctions must be measured accurately on imaging.

Breau *et al.* reported proximal stent migration in 2% of patients in their series and suggested that it occurs when a stent is too short for the ureter or with prolonged indwelling time. They concluded that a longer stent should be placed if stenting a ureter after the migration has been noticed [11]. In addition, placing the proximal curl into the renal pelvis rather than the calyx offers a lesser risk of stent migration. According to Slaton *et al.*, double-J (DJ) stent of shorter than ideal length, inadequate distal curl, and proximal curl of DJ stent in the upper calyx rather than in the renal pelvis appears to be a significant factor responsible for proximal migration of the stent [12]. Stents with full coil are less vulnerable to migrate than those with J shape, and polyurethane stents have a great memory and are less susceptible to migration than silicone DJ stents with less memory [13, 14]. Several other theories have been proposed accounting for proximal migration of double J stent, including prolonged stent indwelling time, stent movement in conjugation with kidney movement during respiration, hydronephrosis, and ureteric calculus alongside the stent acting as a jack- allowing only proximal migration of stent during respiration [15, 16].

Proximally migrated DJ stent rarely causes symptoms; however, it might obstruct urine outflow. Hence, its repositioning or removal is warranted. Literature has described different approaches such as ureteroscopy with the forceps, basket, and ureteral dilators [17–20]. In an analysis of 37 patients, the retrieval of the stents was performed using ureteroscopy with a 91.9% success rate [21]. 

### Irritative symptoms

Irritative symptoms associated with stent placement such as increased urinary urgency or frequency, incontinence, hematuria, bladder pain, and renal colic are very common and significantly decrease quality of life [13, 22]. Similar to our data, irritative bladder symptoms were the most common (42.5%) stent-related complication reported in a study published by M Gurram *et al.* [9]. Other literature data showed that 32.5% of patients with double J stent present important irritative bladder symptoms [23]. Urinary symptoms and pain related to double J ureteral stents can impact daily activities and reduce the quality of life of 60% of patients [24].

Position and completeness of the lower loop influence symptom severity, and stents crossing the midline in the bladder with incomplete loops at the lower end give rise to higher morbidity [25]. According to Gurram’s research, the stent removal rate for fever was 26.3% and 15.6% for irritative bladder symptoms, in contrast to Pansota *et al.*, who found out that the stent removal rate for irritative symptoms was 23.1% and 37.5% for fever [23]. The stent removal rate for fever in the study of Richter *et al.* was very high (55.8%) [26]. However, in our study, stent removal was imposed in only 37 cases from the total of 16,326 patients with irritative bladder symptoms.

### Flank pain

Lumbar pain is also a frequent complication, 19.3% of the patients complaining of it in our study, similar to other data from the literature (19–32%) and seems to appear as a result of urine reflux towards the kidney with an excessive rise in intrapelvic pressure that ultimately translates into pain [27, 28]. The lumbar pain of patients with ureteral stents appears during the voiding of the bladder and seldom during vesical filling [29]. According to Smedley *et al.*, it is usually mild to moderate, and it is not influenced by the position of the proximal coil either in the upper calyx or in the renal pelvis [28].

### Hematuria

An immediate postoperative complication, hematuria, may result from surgical management of existing disease and the stent placement itself [30]. Our data regarding hematuria revealed a 10.4% compared with the incidence found in the literature 13.3%–27.5% [23]. Moreover, in a significant number of cases, hematuria could be secondary to ureteroscopy.

### Encrustation

The encrustation of forgotten stents that have a considerable stone burden is a significant problem. Long-term stenting, urinary tract infection, a history of stone disease, oncologic treatment, chronic kidney failure, and metabolic or congenital defects all favor stent encrustation [31]. It occurs in association with the presence of urease-producing bacteria. These bacteria cause an increase in urine pH, promoting crystal formation. Also, urine composition and pH, stent’s material and surface properties, indwelling time, and urine flow dynamics affect stent encrustation. Although the best time to remove an indwelling ureteral stent is unknown, long-term ureteral stent insertion may increase the risk of stent encrustation or stone development [2, 27]. According to El-Faqih *et al.*, encrustation increased directly to stent indwelling time, which was evident in 76.3% of patients after the 12^th^ week [32]. Similarly, Kawahara *et al.* discovered similar effects in 75.9% of patients after the third month [33]. According to Bultitude MF *et al.*, whether a stent is left in place for 6 weeks, 6–12 weeks, or more than 12 weeks, the rates of stent encrustation are 9.2 percent, 47.5 percent, and 76.3 percent, respectively. Gurram M *et al.* found that the average indwelling time was 38.4 months, much longer than Lam JS *et al.*’s (10.7) and Aravantinos *et al.*’s (24.1) studies. In Gurram’s study, 27 (22.5%) patients had retained double J stents, with 6 (22.2%) having none or minor encrustation. The most common sites of encrustation in the 21 patients with considerable DJS encrustation were the bladder alone (23.8%) and the kidney plus bladder (23.8%) [9, 34, 35].

More severe complications are observed with forgotten catheters at the urinary system that encrust, form stones, contributing to obstruction and loss of renal function [36, 37]. The literature shows that the median time for complications of the indwelling catheter at the urological system is 3–24 months [38]. El-Faqih showed that the rate of complications was 9.6% when the catheter was removed up to 6 weeks following implantation [37]. When maintained for 6 to 12 weeks, this rate rises to 47.5% and 76.3% if this time increases. Kawahara observed similar figures; incrustation was observed in 26.8% before 6 weeks and reached 75.9% when maintained for more than 12 weeks [37]. According to Asem Ali *et al.*, patients with indwelling double J stents for more than 3 months were more susceptible to stent encrustation and stone formation [24, 38]. Often, stents are forgotten either because of patients’ non-compliance who ignore or forget the physician’s advice regarding its timely removal. Poor compliance of the patients was the most common reason for forgotten stents. Encrustation and the resulting stone burden, affecting the bladder, ureter, and kidney, frequently necessitate various endourological methods. It may be necessary to remove the encrusted stents and associated stone burden in single or numerous sessions or open surgery in exceptional cases [39]. A predominance of encrustation at the stent’s upper coil could be explained by the fact that the stent’s lower coil has more effective peristalsis, which sweeps any deposits of the stent, decreasing encrustation [40].

Urologists face a complex problem in managing ureteral stents that have encrustation. The location of encrustation determines the technique of treatment, the amount of the stone burden, and the function of the affected kidney [41]. ESWL has been reported to be a non-invasive and effective first-line therapy for encrustations at the upper coil and/or stent body [42]. Ureteroscopy with pneumatic or ultrasonic lithotripsy can also be used as a first-line treatment or after ESWL failure [43]. A minimally invasive therapeutic option is flexible ureteroscopy with holmium laser lithotripsy [44]. More invasive procedures, such as PCNL or open pyelolithotomy, are frequently required in heavily encrusted stents. To decrease the number of surgeries and associated morbidity, it is necessary to assess the exact size and location of the stone burden prior to surgery, establish where the stent is trapped, and then manage it accordingly. The availability of technology, on the other hand, influences therapy selection.

Extracorporeal shock wave lithotripsy (ESWL) may be utilized to treat stents with minor encrustation. In the Mathew *et al.* trial, 14 (29.1%) patients required ESWL, with 10 (20.8%) additionally requiring ureteroscopy [45]. According to Matthew *et al.*, 38 (79.1%) patients required URS, with 28 (58.3%) requiring only URS, 5 (10.4%) patients requiring percutaneous nephrolithotomy (PCNL), and 1 (2.1%) patient requiring open stent removal. Upper coil PCNL can be used in patients with a significant stone burden. Open surgery is performed in individuals who have had their endourological treatment fail or have a substantial stone burden [2].

All 6 (22.2%) patients with residual double J stents, with minimum or no encrustation, were treated by cystoscopic removal of the stent under local anesthesia in Gurram’s study. The remaining 21 patients with a double J stent that had encrustation required one or more procedures. Per patient, the average number of procedures was 1.71. Sixteen patients (76.19%) required CLT; 5 patients (23.8%) required CLT alone, while 11 patients (68.75%) required supplementary PCNL, URS, or both. A total of 7 patients (33.33%) required ureteroscopy in conjunction with other operations, whereas 9 (42.87%) required PCNL. Each patient needed a pyelolithotomy and a nephrectomy. In this study, two patients (9.52%) developed sepsis after surgery, necessitating intensive care and broad-spectrum antibiotics, although no fatality was seen [9].

Borboroglu *et al.* also described the endourological therapy of four patients who had severely encrusted ureteral stents and a high stone burden. All patients required two to six endourological procedures (average 4.2) conducted in one or several sessions to attain stone-free and stent-free status [41]. In ten patients, Bukkapatnam R *et al.* reported one-stage removal of 12 encrusted retained ureteral stents [46].

A multimodal approach is often necessary to treat the forgotten ureteral stent with a significant stone burden [2]. Many series reported an average of 2.7 and 2.38 procedures for clearing their patients from retained stents and the associated stones [35]. Bostanci *et al.* used a variety of endourological procedures in 19 patients with encrusted ureteral stents and removed the stents and stones in a single anesthesia session with low morbidity and a short hospital stay [47].

In our study, following the volume and localization of the encrustation/calcification, the treatment alternative was represented by simple double J stent removal in 241 cases, lithotripsy of the bladder loop calcifications (324 cases), lithotripsy of the bladder loop calcifications associated with PCNL (129 cases), lithotripsy of the bladder loop calcification and retrograde ureteroscopy (115 cases), PCNL alone (11 cases) and retrograde ureteroscopy (12 cases). Avoiding excessive force for stent extraction is always imperative. Using the force can produce serious complications like ureteral injuries or avulsion [48]. 

### Stent fragmentation

Fragmentation is a major consequence of forgotten double J stents [48]. This happens when the stent loses its tensile strength when the polymers degrade, and the stent hardens [48, 49]. The rate of ureteral stent fragmentation is zero. In the literature, 3–10% is mentioned [37, 48].

Encrustation in forgotten stents is occasionally linked to stent breakage. Due to hardening and loss of tensile strength, stents may spontaneously fracture after being in place for a long time [50].

## Conclusions

Double J stents are a valuable tool for urologists to prevent and alleviate blockage. Unfortunately, there is no such thing as a “perfect urinary stent”, and these are not without risks. Complications of the Double J stent should be assessed and addressed as soon as possible. Patients should be advised to remove their devices as soon as possible and to change them regularly if necessary to remain chronically indwelling. Encrustation and stone formation in forgotten stents can cause life-threatening issues; thus, they should be removed using a combination of endourologic methods.

## Acknowledgments

### Conflict of interest

The authors declare that there is no conflict of interest.

### Consent to participate

Written informed consent was obtained from the participants.
